# 2282 Targeted eccentric motor control to improve locomotion after incomplete spinal cord injury

**DOI:** 10.1017/cts.2018.126

**Published:** 2018-11-21

**Authors:** Kevin O’Brien, D. Michele Basso, James Schmiedeler

**Affiliations:** Indiana University School of Medicine

## Abstract

OBJECTIVES/SPECIFIC AIMS: Incomplete spinal cord injury typically results in life-long disability, often in the form of profound loss of locomotion capability. Individuals who have experienced incomplete spinal cord injury exhibit persistent eccentric motor deficits, which are particularly prevalent in the weight acceptance phase of gait and emphasized in sagittal plane knee motion and frontal plane hip motion. METHODS/STUDY POPULATION: Motion analysis can capture the kinematic and joint-level deficits of these individuals, but it is impossible to directly calculate the contributions of individual muscles to weight acceptance due to the complexity of the musculoskeletal system. Instead, those muscle contributions must be simulated in order to approximate muscle power during locomotion. RESULTS/ANTICIPATED RESULTS: The traditional method for driving these simulations with electromyography readings is unavailable for individuals who have neuromuscular deficits (e.g., spasticity or paralysis), due to the need to generate reliable maximum voluntary isometric contractions for baseline purposes. Instead, this research develops a novel method for using resting electromyography data to drive musculoskeletal simulations using a muscle activation threshold paradigm. DISCUSSION/SIGNIFICANCE OF IMPACT: The simulation results of this method more closely resemble experimental results, but further simulation refinement is needed to fully capture the true muscle activity.

